# Convenient preparation of high molecular weight poly(dimethylsiloxane) using thermally latent NHC-catalysis: a structure-activity correlation

**DOI:** 10.3762/bjoc.11.246

**Published:** 2015-11-20

**Authors:** Stefan Naumann, Johannes Klein, Dongren Wang, Michael R Buchmeiser

**Affiliations:** 1 Institute for Polymer Chemistry, University of Stuttgart, Pfaffenwaldring 55, D-70569 Stuttgart, Germany; 2 Institut für Chemie und Biochemie, Anorganische Chemie, Freie Universität Berlin, Fabeckstraße 34–36, Berlin, Germany; 3 Institute of Textile Chemistry and Chemical Fibers, Körschtalstrasse 26, D-73770 Denkendorf, Germany

**Keywords:** latency, N-heterocyclic carbenes, ring-opening organocatalysis, polymerization, polysiloxanes

## Abstract

The polymerization of octamethylcyclotetrasiloxane (D_4_) is investigated using several five-, six- and seven-membered *N*-heterocyclic carbenes (NHCs). The catalysts are delivered in situ from thermally susceptible CO_2_ adducts. It is demonstrated that the polymerization can be triggered from a latent state by mild heating, using the highly nucleophilic 1,3,4,5-tetramethylimidazol-2-ylidene as organocatalyst. This way, high molecular weight PDMS is prepared (up to >400 000 g/mol, 1.6 < *Ð*_M_ < 2.5) in yields >95%, using low catalyst loadings (0.2–0.1 mol %). Furthermore, the results suggest that a nucleophilic, zwitterionic mechanism is in operation, in preference to purely anionic polymerization.

## Introduction

N-Heterocyclic carbenes (NHCs) [1–3] have had a resounding impact on organopolymerization [4–5] during the past fifteen years. Considerable research effort has steadily deepened the mechanistic understanding of the polymerization pathways open to NHCs, while the range of accessible monomer structures has grown impressively [6–7]. Nowadays, NHC-mediated polymerization can be applied to prepare polymers of high industrial and commercial importance, such as poly(amide)s [8–9], poly(ether)s [10], poly(urethane)s [11–12] or poly(acrylate)s [13–18]. Likewise, poly(siloxane)s are attractive and versatile macromolecular materials produced on large scale and thus a rewarding field for the development of new catalysts, the more so if the added benefit of metal-free conditions can be implemented [19–21]. In spite of this, few investigations regarding the performance of NHCs in this area have been published. In 2006, Waymouth, Hedrick and co-workers showed that poly(carbosiloxane)s can be synthesized efficiently from the monomer 2,2,5,5-tetramethyl-1-oxa-2,5-disilacyclopentane in the presence of alcohols as initiators, using two different NHCs [22]. Control over the molecular weight was good (*Ð*_M_ < 1.2), but prolonged polymerization led to transetherification. Interestingly, the formation of high molecular weight cyclic poly(carbosiloxane) was observed in the absence of an initiator [23]. Additionally, octamethylcyclotetrasiloxane (D_4_) was used to prepare poly(dimethylsiloxane) (PDMS), using three different imidazolium-based free NHCs in combination with benzyl alcohol or methanol as initiator [24]. There, polymerizations were conducted at 80 °C for 16 h to achieve conversions of about 85% (1.5 < *Ð*_M_ < 1.7, 0.1% catalyst loading). Notably, it was found that steric hindrance of the NHC shut down polymerization activity. Finally, a report on the polycondensation of α,ω-disilanols describes the efficient polycondensation via NHCs in spite of the generation of water during the reaction, finding that the most basic NHC in the small study delivered the best results [25]. In view of these promising, yet somewhat limited, results, the aim for this study was (a) to investigate a range of structurally diverse NHCs to get more insight into the influence of NHC structure on catalytic activity for the polymerization of D_4_, and (b) to generate the NHCs in situ from thermally labile CO_2_ adducts. This type of NHC delivery offers the double advantage of improved stability and storability of the NHC adduct and the possibility to generate “on demand” polymerization systems where the catalyst can be activated by heating. This way, a latent, easy-to-handle metal-free process which is more competitive in comparison with other catalytic systems can be realized [26].

## Results and Discussion

**Scheme 1:**
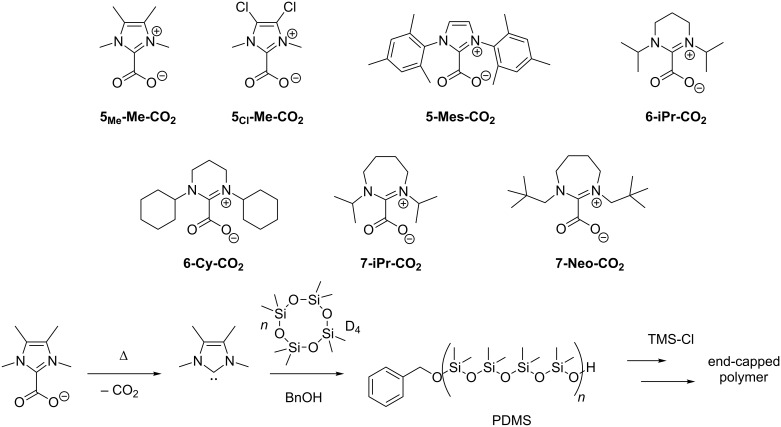
NHC-carboxylates part of this study (top) and polymerization scheme with initial thermal decarboxylation and final end-capping with TMS-Cl.

Several five-, six- and seven-membered NHCs were prepared and reacted with carbon dioxide to receive the corresponding CO_2_ adducts (Scheme 1, top), following literature procedures (see Supporting Information File 1). Compounds **7-Neo-CO_2_**, **7-iPr-CO_2_** and **5_Cl_-Me-CO_2_** have not been described before and full characterization can be found in the experimental part (Supporting Information File 1). In accordance with previous findings [17,27], these NHC-carboxylates were stable at room temperature over long periods of time (observed for up to 2 years) without decomposition, exclusion of humidity provided.

Polymerization experiments with D_4_ were conducted solvent-free at elevated temperature (80 °C, 16 h), both in presence and absence of an initiator (benzyl alcohol, BnOH). This immediately revealed sharp differences of reactivity between the individual pre-catalysts (Table 1). When the protected NHC was applied in conjunction with BnOH, only **5_Me_-Me-CO_2_** showed relevant activity, albeit a very high one.

**Table 1:** Polymerization of D_4_ in bulk using different protected NHCs (80 °C, 16 h).

Entry	NHC-CO_2_	NHC/BnOH/D4	Conversion [%]^a^	*M*_n_ ×10^3^ [g/mol]^b^	*Ð*_M_

1	**5_Me_-Me-CO_2_**	1:5:500	94	70	1.7
2	**5_Me_-Me-CO_2_**	1:1:100	>95	198	1.9
3	**5_Me_-Me-CO_2_**	1:1:300	>95	288	1.9
4	**5_Me_-Me-CO_2_**	1:1:700	>95	360	2.5
5	**5_Me_-Me-CO_2_**	1:1:1000	>95	424	2.2
6	**5-Mes-CO_2_**	1:5:500	7	–	–
7	**5_Cl_-Me-CO_2_**	1:5:500	0	–	–
8	**6-iPr-CO_2_**	1:5:500	0	–	–
9	**6-Cy-CO_2_**	1:5:500	0	–	–
10	**7-iPr-CO_2_**	1:5:500	5	–	–
11	**7-Neo-CO_2_**	1:5:500	0	–	–
12	**5_Me_-Me-CO_2_**	1:0:100	92	8	1.3
13	**6-Cy-CO_2_**	1:0:100	insoluble	n. d.	–
14	**7-iPr-CO_2_**	1:0:100	insoluble	n. d.	–

^a^Determined by ^1^H NMR spectroscopy; ^b^via GPC (THF, PS standards).

**Figure 1:**
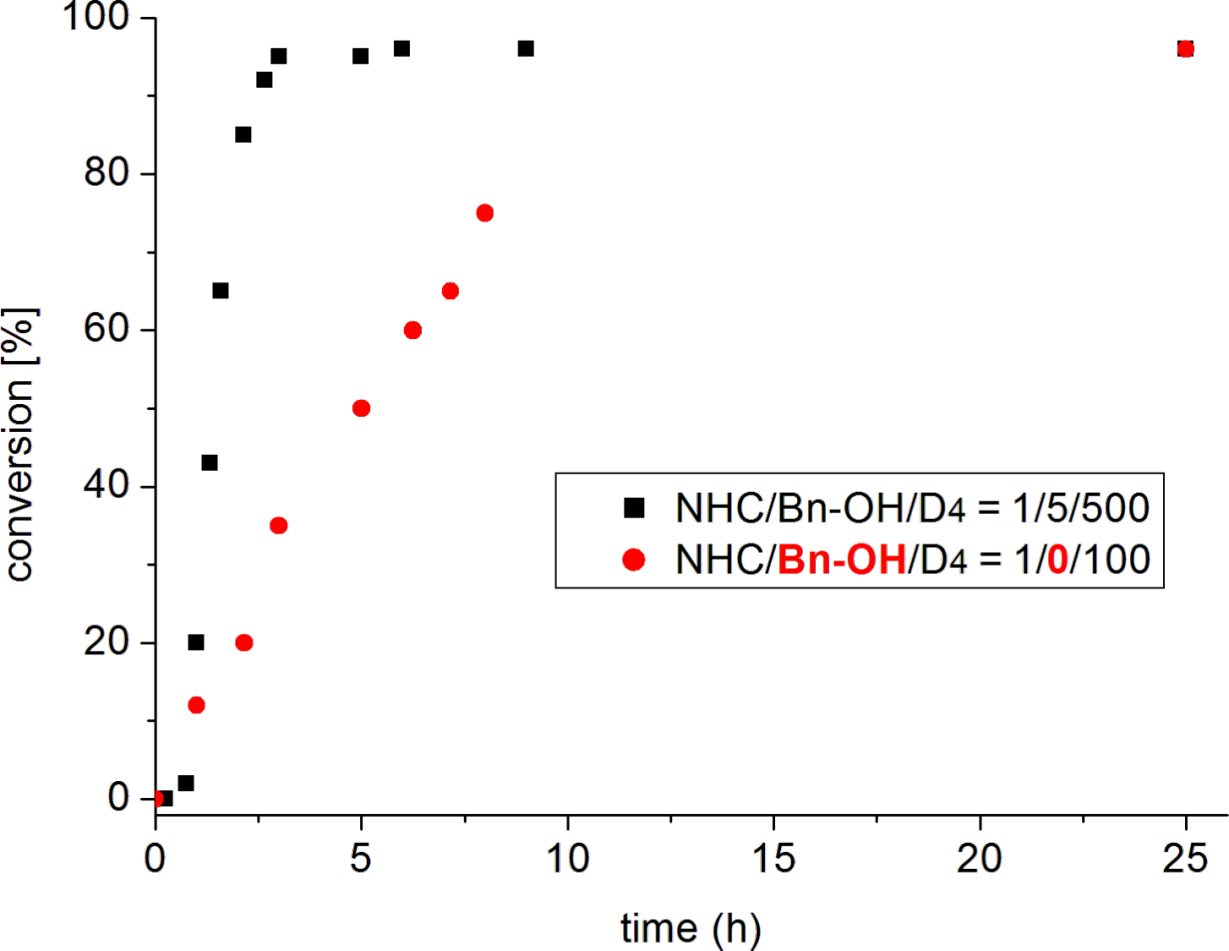
Comparison of conversion over time for D_4_ polymerization (80 °C, bulk) using **5_Me_-Me-CO_2_**. Note that the fivefold monomer excess was used in case of BnOH being present.

At a catalyst loading of only 0.2 mol %, a molecular weight (*M*_n_) of 70 000 g/mol was achieved at 94% monomer conversion. Interestingly, some degree of control over the molecular weight is possible by adjusting the initiator to monomer ratio (Table 1, entries 2–5). This way, up to a target degree of polymerization (DP) of 1000, considerable molecular weight can be built up, ranging from 200 000 g/mol to over 400 000 g/mol (*M*_n_). Importantly, in all these cases very high conversion is observed. Matrix-assisted laser desorption/ionization time-of-flight mass spectrometry (MALDI–ToF MS) and NMR experiments clearly show that BnOH is incorporated in the resulting PDMS (S1–S3), underlining the defined structure of the polymer. Contrasting this behaviour of **5_Me_-Me-CO_2_**, its sterically more hindered analogue, **5-Mes-CO_2_**, only brought about a low conversion of 7% under identical conditions (Table 1, entry 6), while its electron-poor derivative **5_Cl_-Me-CO_2_** was completely inactive (Table 1, entry 7). More surprisingly, the six- and seven-membered pre-catalysts were all found to deliver very little or no polymer. Differently, in the absence of BnOH (1% catalyst loading, Table 1, entries 12–14), application of both **7-iPr-CO_2_** and **6-Cy-CO_2_** resulted in an insoluble polymer, most probably because very high molecular weight was generated. Gratifyingly, under the same parameters **5_Me_-Me-CO_2_** effected a conversion of 92% (*M*_n_ = 8 100 g/mol). Overall, polymerization with this pre-catalyst proceeds noticeably faster in the presence than in the absence of an initiator (Figure 1). At a polymerization setup of NHC/BnOH/D_4_ = 1:5:500, the conversion is practically complete after only 2.5 h.

Above findings obviously render **5_Me_-Me-CO_2_** the most suitable pre-catalyst, but important conclusions with regard to the polymerization mechanism can also be drawn. In part, the results nicely mirror findings by Baceiredo and co-workers [24], who described that steric hindrance eliminates any polymerization activity of the NHC; the same is found here when comparing the performance of **5_Me_-Me-CO_2_** and **5-Mes-CO_2_**. Additionally, electron-withdrawing substituents (as present in **5_Cl_-Me-CO_2_**) preclude any activity, emphasizing that nucleophilicity is crucial for successful polymerization. Furthermore, it is interesting to note that the six- and seven-membered NHCs do not show any reactivity here, in spite of being very strong bases. While for a compound like **6-iPr** a p*K*a-value of 28.2 (aqueous solution, 25 °C) was found, the five-membered imidazolium derivative **5-Mes** was determined to have a p*K*a-value of only 20.8 (which compares to 15–19 for typical alcohols; in turn, silanols are even more acidic than the corresponding alcohols) [28–32].

At the same time, **5_Me_-Me-CO_2_** successfully catalyzed PDMS-formation in the absence of BnOH (see above), and hence a mechanism for direct D_4_ polymerization must exist.

**Scheme 2:**
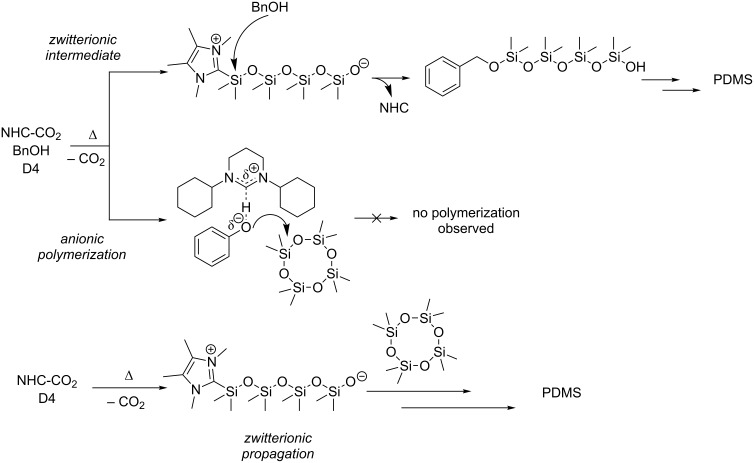
Discussed mechanisms proposed to operate in NHC-mediated polymerization of D_4_ in presence/absence of BnOH.

Taken together, all above points strongly suggest that nucleophilic ring-opening of the monomer by the NHC is the key step, in agreement with previous proposals [24]. A purely basic (anionic) pathway (Scheme 2), often preferred by the six-membered NHCs [7–9,17], seems clearly disfavoured. In the absence of BnOH, it is therefore reasonable to assume zwitterionic propagation, in analogy to recent findings for NHC-mediated lactone polymerization [33]. The insoluble material received by the action of **7-iPr-CO_2_** and **6-Cy-CO_2_** could be connected to a low initiation efficiency and consequently low ratio of propagating zwitterions to monomer, resulting in high-molecular weight polymers. In contrast, a sterically uncongested, highly nucleophilic NHC as liberated from **5_Me_-Me-CO_2_** (Table 1, entry 12) is more suitable under these conditions.

**Figure 2:**
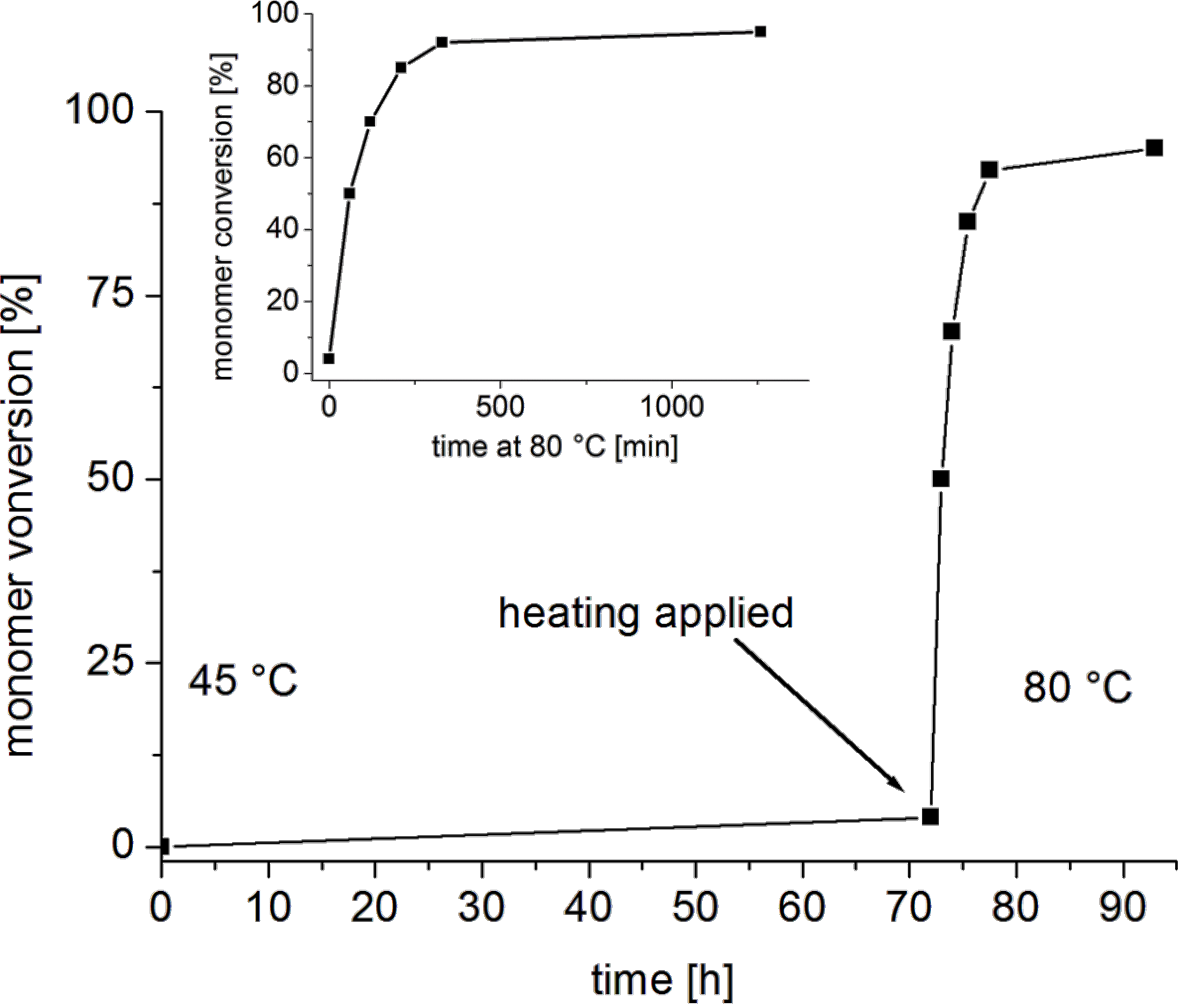
Thermal activation of a **5_Me_-Me-CO_2_**/BnOH/D_4_ (1:5:500) composition after a latency period of 72 h.

Finally, a mixture of **5_Me_-Me-CO_2_**, BnOH and D_4_ was tested for thermal latency (Figure 2). Notably, after 72 h at a slightly elevated temperature of 45 °C only negligible conversion was observed. A relatively mild increase to 80 °C, however, triggered a clean jump to near quantitative conversion. Hence, with this catalytic setup it is possible to form one-component, metal-free mixtures which can be activated by mild heating. Polymerization of D_4_ is entropically driven [21,34] and thus profits from elevated temperature in any case, rendering the implementation of thermally labile pre-catalysts both practically feasible and advantageous.

## Conclusion

In conclusion, we have demonstrated the first polymerization of D_4_ using NHC-carboxylates. Sterically non-hindered, highly nucleophilic protected NHCs like **5_Me_-Me-CO_2_** offer access to an “on demand”, metal-free and effective preparation of PDMS, including high molecular weight polymers. The results of a screening of a range of different NHCs indicate that a nucleophilic action of the organocatalyst is preferred over action as a Brønsted base.

## Supporting Information

File 1 Details on the synthesis of NHC-CO_2_ and polymerizations.
