# Supplementary material for: Convenient preparation of high molecular weight poly(dimethylsiloxane) using thermally latent NHC-catalysis: a structure-activity correlation
Source: Beilstein J Org Chem. 2015 Nov 20;11:2261–6. doi: 10.3762/bjoc.11.246 (PMC4660969; doi:10.3762/bjoc.11.246)
Supplement: File 1 — Details on the synthesis of NHC-CO2 and polymerizations. [file Beilstein_J_Org_Chem-11-2261-s001.pdf]

Supporting Information

for

Convenient preparation of high molecular weight poly(dimethylsiloxane) using thermally latent NHC-catalysis: a structure-activity correlation

Stefan Naumann[1], Johannes Klein[2], Dongren Wang[1] and Michael R. Buchmeiser[1,3*]

Address: [1]Institute for Polymer Chemistry, University of Stuttgart, Pfaffenwaldring 55, D-70569 Stuttgart, Germany, [2]Institut für Chemie und Biochemie, Anorganische Chemie, Freie Universität Berlin, Fabeckstraße 34–36, Berlin, Germany and [3]Institute of Textile Chemistry and Chemical Fibers, Körschtalstrasse 26, D-73770 Denkendorf, Germany

Email: Prof. Dr. Michael R. Buchmeiser – michael.buchmeiser@ipoc.uni-stuttgart.de

* Corresponding author

Details on the synthesis of NHC-CO_2 and polymerizations

Contents

Experimental S2
Materials and synthesis S2
Polymerizations S3
Characterization and analysis S3
MALDI–ToF MS and NMR S5
References S7

Experimental

Materials and synthesis

Air- and moisture sensitive preparations and polymerizations were carried out using standard Schlenk techniques or in a nitrogen filled glove box (MBraun Labmaster 130). For moisture sensitive applications, CH_2Cl_2, diethyl ether, toluene, pentane and THF were dried using a solvent purification system (SPS800, MBraun). Deuterated dichloromethane was dried over calcium hydride, followed by subsequent distillation under nitrogen and degassing. Benzyl alcohol was dried over a small amount of sodium, distilled under nitrogen and degassed. Octamethylcyclotetrasiloxane (D_4, ABCR or Alfa Aesar) was stored over molecular sieves in a glove box. For synthesis and full characterization of **5_{Me}-Me-CO_2**,[i] **5-Mes-CO_2**,[ii] **6-iPr-CO_2**,[iii] and **6-Cy-CO_2**,[iv] see the cited literature.

1,3-Diisopropyltetrahydro-[1,3]-diazepinium-2-carboxylate (7-iPr-CO_2). 1,3-Diisopropyltetrahydro-[1,3]-diazepinium bromide[v] (550 mg; 2.10 mmol) was suspended in THF (15 mL). KO*t*-Bu (282.55 mg; 2.51 mmol) was dissolved in THF and added to the suspension. The mixture was stirred for 45 min at room temperature, the solvent was removed in vacuo and the remaining solid was extracted with diethyl ether and filtered. CO_2 was bubbled through the yellowish solution for 5 min. The thereby precipitated colorless solid was separated, washed with diethyl ether and dried in vacuo (120 mg; 0.53 mmol; 25% yield). **^{1}H NMR (400 MHz, CD_2Cl_2):** δ = 4.32 (sep, ^3J=6.6 Hz, 2H), 3.49 (m, 4H), 2.0 (m, 4H), 1.26 (d, ^3J=6,6 Hz, 12H) ppm. **^{13}C NMR (100 MHz, CD_2Cl_2):** δ = 168.4, 160.7, 55.2, 42.9, 25.1, 20.3 ppm. **IR (ATR, cm^{-1}):** 2963 (w), 1653 (s), 1574 (s), 1297 (s), 1266 (m), 1161 (s), 976 (m), 813 (s), 792 (m); 738 (s). **HRMS (ESI):** *m/z* calc. for $C_{12}H_{22}N_2O_2$: 226.1681; found 249.1559 $[M + Na]^+$.

1,3-Bis(2,2-dimethylpropan)tetrahydro-[1,3]-diazepinium-2-carboxylate (7-Neo-CO_2). 1,3-Bis(2,2-dimethylpropan)tetrahydro-[1,3]-diazepinium bromide[v] (1.2 mg; 3.80 mmol) was suspended in THF (25 mL). KHMDS (758 mg; 3.80 mmol) was dissolved in THF and added to the suspension. The mixture was stirred for 45 min at room temperature, the solvent was removed in vacuo and the remaining solid was extracted with diethyl ether and filtered. CO_2 was bubbled through the yellowish solution for 5 min. The thereby precipitated colorless solid was separated, washed with diethyl ether and dried in vacuo (175 mg; 0.62 mmol; 16% yield). **^{1}H NMR (400 MHz, CD_2Cl_2):** δ = 3.64 (m, 4H), 3.43 (s, 4H), 2.02 (m, 4H), 1.04 (s, 18H) ppm. **^{13}C NMR (100 MHz, CD_2Cl_2):** δ = 173.0, 160.0, 68.9, 56.3, 33.1, 28.5, 25.2

ppm. **IR (ATR, cm^{-1}):** 2955 (s), 1657 (s), 1570 (s), 1458 (m), 1400 (m) 1294 (s) 1265 (s), 1025 (s), 895 (s), 848 (m), 791 (s). **HRMS (ESI):** *m/z* calc. for $C_{16}H_{30}N_2O_2$: 282.2307; found 305.2199 $[M + Na]^+$.

4,5-Dichloro-1,3-dimethylimidazolium-2-carboxylate (5_{Cl}-Me-CO_2). 4,5-Dichloro-1,3-dimethylimidazolium iodide[vi] (253 mg; 0.86 mmol) was suspended in THF (10 mL). To this, KHMDS (172 mg; 0.86 mmol) dissolved in THF (2 mL) was dropped. After 45 minutes stirring at room temperature, the solvent was removed in vacuo and the residues extracted with diethyl ether. After filtration, an orange-colored solution was received. Treatment with CO_2 resulted in the precipitation of an orange solid. This was separated and washed with diethyl ether, then dried under vacuum (90 mg, 0.43 mmol, 50%). **^{1}H NMR (400 MHz, CD_2Cl_2):** δ = 4.12 (s) ppm. **^{13}C NMR (100 MHz, CD_2Cl_2):** δ = 153.4, 143.9, 119.2, 35.4 ppm. **IR (ATR, cm^{-1}):** 2958 (w), 1673 (s), 1578 (m), 1494 (m), 1308 (s), 1155 (m), 808 (s), 601 (m), 435 (m). **HRMS (ESI):** *m/z* calc. for $C_6Cl_2H_6N_2O_2$: 207.98; found: 165.00 $[M - CO_2 + H]^+$.

Polymerizations

For the polymerization of D_4 (500–2000 mg), the desired amount of the CO_2-protected NHC and (if required) benzyl alcohol were combined in a Schlenk tube containing a magnetic stirrer. The sealed tube was then removed from the glovebox and placed in a pre-heated oil bath (80 °C). After the reaction (typically 16 h, high viscosity), the polymerization was stopped by adding trimethylsilyl chloride (TMS-Cl) and dissolved in chloroform. The PDMS was then precipitated from methanol and dried under vacuum.

Conversion was determined via ^{1}H NMR spectroscopy ($CDCl_3$), using the distinct signals of polymer and monomer at δ = 0.07 and 0.09 ppm, respectively. The molecular weight of the PDMS was analyzed by GPC (see below). For kinetic measurements, aliquots were taken from the reaction mixture to determine the conversion by ^{1}H NMR spectroscopy, using heat-dried pipettes (data for Figures 1 and 2).

Characterization and analysis

NMR measurements were recorded on a Bruker Avance III 400 spectrometer. All spectra were analyzed using reference peaks for ^{1}H and ^{13}C NMR experiments of $CDCl_3$ (δ = 7.26 ppm/77.16 ppm) or CD_2Cl_2 (δ = 5.32 ppm/54 ppm), respectively. IR spectra were measured on a Bruker Alpha FTIR spectrometer (4000–400 cm^{-1}). GPC (THF) measurements were

conducted against polystyrene calibration on a chromatographic system consisting of a Waters 515 HPLC pump, a Waters 2707 autosampler and PolyPore columns (Agilent), in combination with a Waters 2414 refractive index detector. The flow rate was 1 mL/min (room temperature) using a sample concentration of 3 mg/mL. For MALDI–ToF MS analysis, measurements were performed on a Bruker Autoflex III with a smart ion beam laser (337 nm). Measurements were carried out in reflector mode. Samples were prepared by mixing matrix solution (2,5-dihydroxybenzoic acid, 0.1 M in acetone), polymer solution (10 mg/ml in CH_2Cl_2), and NaI solution (0.05 M in 90% acetone, 10% water) with a ratio of 1:0.5:1. For calibration, a polystyrene standard was applied (1900 Da).

MALDI–ToF MS and NMR

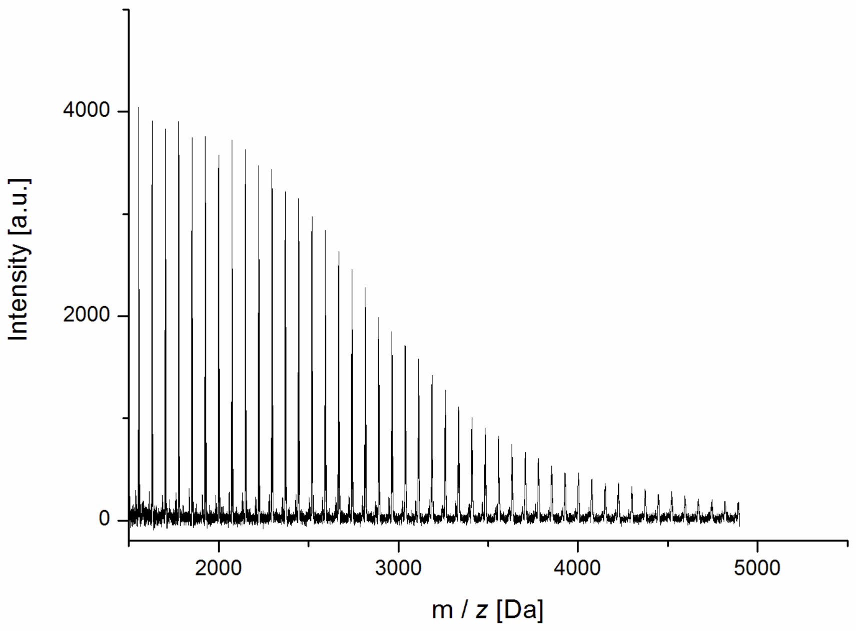

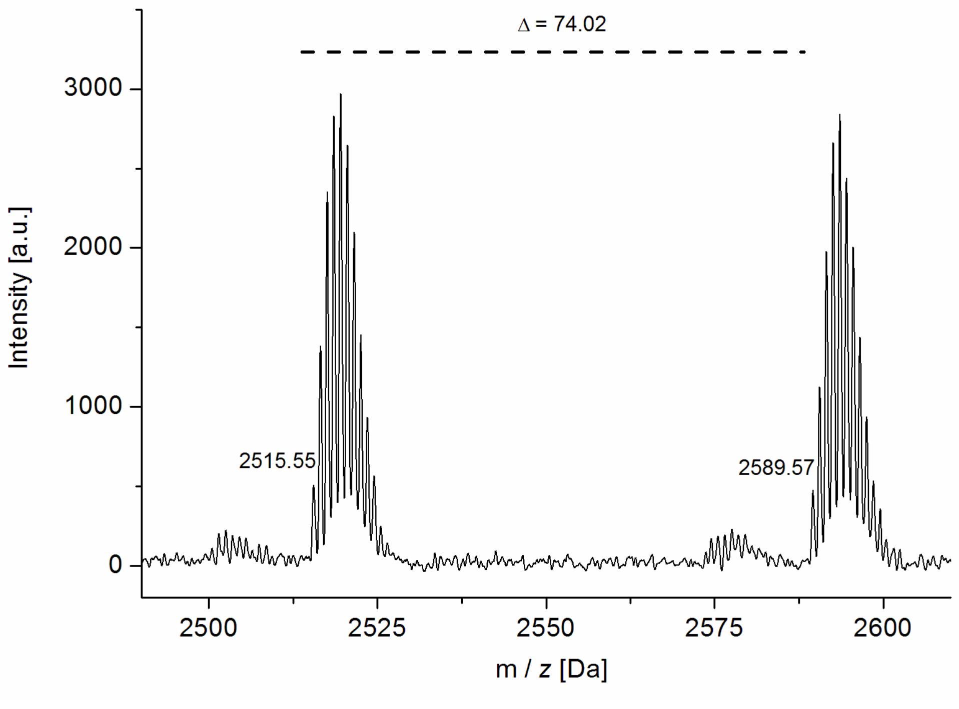

Figure S1: (Top) Full MALDI–ToF MS spectrum of a PDMS sample derived by the action of **5_{Me}-Me-CO_2** (NHC/BnOH/D_4 = 1:50:250, 16 h, 80 °C, no TMSCl). (**Bottom**) Detail showing the experimentally found repeating unit (calc. for [-Si$(CH_3)_2$-O-]: 74.02).

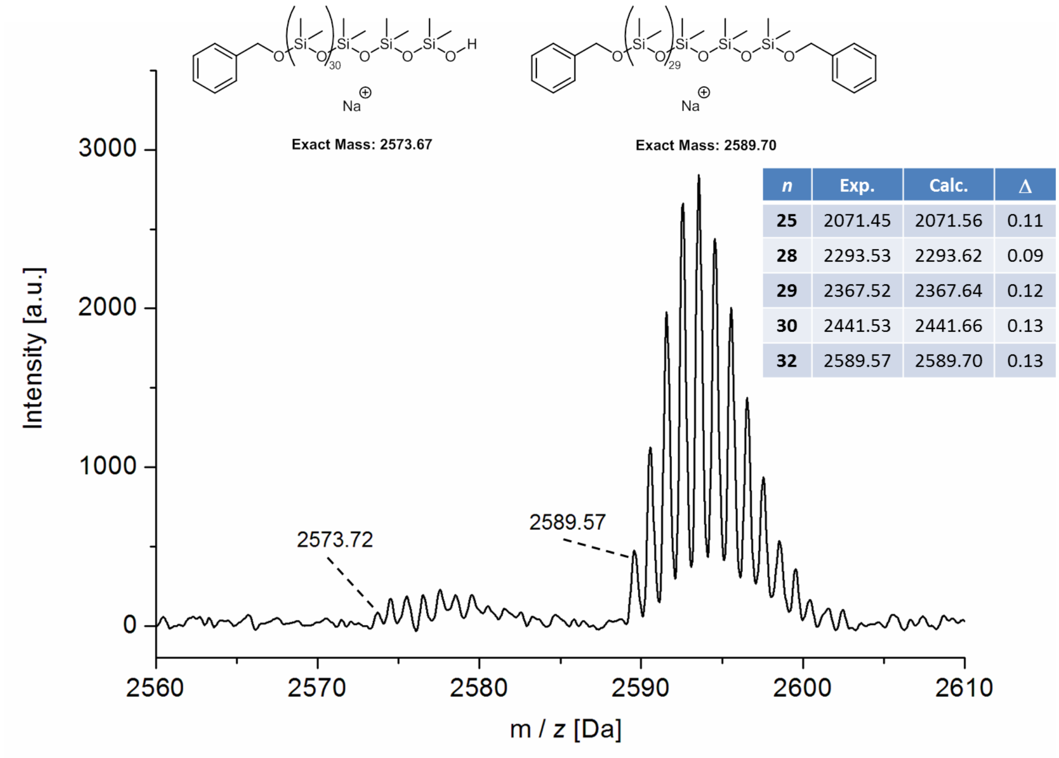

n	Exp.	Calc.	Δ
25	2071.45	2071.56	0.11
28	2293.53	2293.62	0.09
29	2367.52	2367.64	0.12
30	2441.53	2441.66	0.13
32	2589.57	2589.70	0.13

Figure S2: Detail of the same polymer (S1), showing the major and minor mass distribution. The minor signal set can be attributed to BnOH-initiated and hydroxy terminated PDMS, while the major distribution results from condensation of –OH terminated polymer. See also the tabulated values for further comparison of experimental and calculated masses.

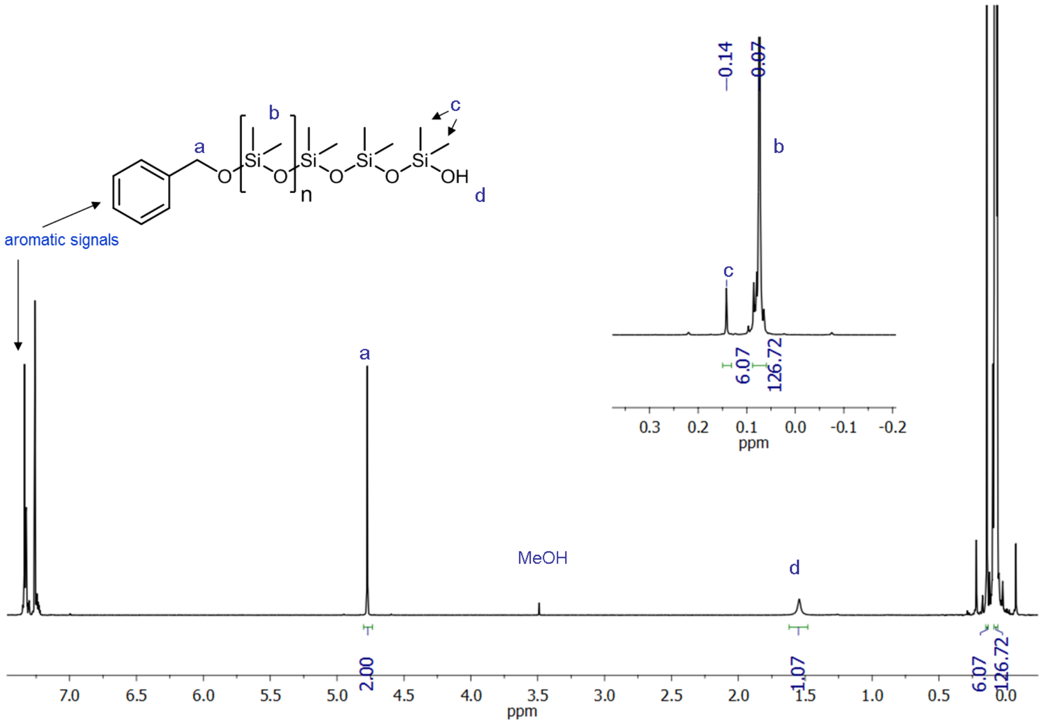

Figure S3: ^{1}H NMR spectrum ($CDCl_3$, 400 MHz, room temperature) of polymer derived from $\mathbf{5_{Me}}$**-Me-**$\mathbf{CO_2}$ (NHC/BnOH/D_4 = 1:50:250, 16 h, 80 °C, no TMSCl) after precipitation from MeOH.

References

[i] a) Van Ausdall, Bret R., J. L. Glass, K. M. Wiggins, A. M. Aarif, J. Louie, *J. Org. Chem.* **2009**, *74*, 7935–7942; b) N. Kuhn, T. Kratz, *Synthesis* **1993**, *1993*, 561–562.

[ii] Duong, H. A.; Tekavec, T. N.; Arif, A. M.; Louie, J. *Chem. Commun.* **2004,** 112–113.

[iii] Bantu, B.; Pawar, G. M.; Decker, U.; Wurst, K.; Schmidt, A. M.; Buchmeiser, M. R. *Chem. Eur. J.* **2009,** *15* (13), 3103–3109.

[iv] Naumann, S.; Schmidt, F. G.; Schowner, R.; Frey, W.; Buchmeiser, M. R. *Polym. Chem.* **2013,** *4*, 2731–2740.

[v] Dunsford, J. J.; Tromp, D. S.; Cavell, K. J.; Elsevier, C. J.; Kariuki, B. M. *Dalton Trans.* **2013,** *42*, 7318–7329.

[vi] Khramov, D. M.; Lynch, V. M.; Bielawski, C. W. *Organometallics* **2007,** *26*, 6042–6049.
